# Causal effects of transitions to adult roles on early adult smoking and drinking: Evidence from three cohorts

**DOI:** 10.1016/j.socscimed.2017.06.018

**Published:** 2017-08

**Authors:** Michael J. Green, Alastair H. Leyland, Helen Sweeting, Michaela Benzeval

**Affiliations:** a MRC/CSO Social & Public Health Sciences Unit, University of Glasgow, 200 Renfield Street, Glasgow, G2 3QB, UK; b Institute for Social and Economic Research, University of Essex, Colchester, CO4 3SQ, UK; c Institute of Health and Wellbeing, University of Glasgow, 1 Lilybank Gardens, Glasgow, G12 8RZ, UK

**Keywords:** Smoking, Alcohol consumption, Transitions, Education, Causal inference, UK

## Abstract

Transitions into work and family roles have become increasingly delayed as participation in tertiary education widens. Such transitions may have adverse or beneficial effects on health behaviours such as smoking and drinking (alcohol). Role socialisation effects may reduce smoking or drinking, but clustering of transitions may lead to role overload, weakening or reversing any role socialisation effects. Effects of transitions were examined in three UK cohorts: the 1958 National Child Development Study, the 1970 British Birth Cohort Study, and the West of Scotland: Twenty-07 Youth Cohort (from around Glasgow, growing up in the same time period as the 1970 cohort). Latent class analysis was employed to identify heterogeneous patterns of transition timing for leaving education, entering employment, starting cohabitation, having a first child, and leaving the parental home. Propensity weighting was then used to estimate causal effects of transition patterns (relative to tertiary education) on smoking and heavy drinking in early adulthood (ages 22–26), adjusting for background confounders (gender, parental socioeconomic position, family structure, parental and adolescent health behaviours, adolescent distress and school performance). Three groups made early (age 16) transitions from education to employment and then either delayed other transitions, made other transitions quickly, or staggered transitions with cohabitation beginning around ages 19–21; a fourth group transitioned from education to employment around ages 17–18. Compared to those in tertiary education with similar background characteristics, those in these groups generally had higher levels of smoking, especially where transitions were more clustered, but less heavy drinking (except those who delayed other transitions after moving into employment). Results partially supported role socialisation effects for drinking, and role overload effects for smoking. Wider participation in tertiary education could have helped reduce smoking levels in these cohorts, but might also have increased risk for heavy drinking.

## Highlights

• Those not in tertiary education experience heterogeneous life transitions.
• Early transitions to adult roles may increase risk of smoking in early adulthood.
• Early entry to adult roles may reduce risk of heavy drinking in early adulthood.

## 1 Introduction

Participation in tertiary education has been growing in the UK since the 1960s (Machin and Vignoles, 2004), and it is important to understand the health effects of this expansion. Many of these may be long term, with education leading to accumulated advantages in socioeconomic and health terms over the lifecourse (Graham, 2007). However, tertiary education primarily occurs as young people transition from adolescence to adulthood, which is an important phase of life for the development of health behaviours (Arnett, 2005, Staff et al., 2010). Young people not in education experience heterogeneous patterns of life transitions such as entering employment, starting cohabiting relationships, or having children; these transitions tend to be delayed by those who participate in tertiary education (Cohen et al., 2003, Côté and Bynner, 2008, Schulenberg and Maggs, 2002). Regardless, this period is characterised by movement into new social environments and networks, where the social functions of smoking and drinking may be particularly valued (Pavis et al., 1998, Schulenberg and Maggs, 2002). New and unfamiliar demands may be experienced as stressful and over-whelming and smoking and drinking behaviours may be used as coping mechanisms (Arnett, 2005, Kuntsche et al., 2005, Schulenberg and Maggs, 2002). Participants in tertiary education often leave the parental home, and associated reductions in parental monitoring of behaviour, along with the delay of responsible adult social roles (like employee, partner or parent; Arnett, 2000, Arnett, 2005) may result in more freedom to smoke and drink. We investigate the impact of late adolescent transitions on young people's smoking and drinking behaviours, comparing those following a range of different non-educational pathways with those who remain in education. We draw below on theories of role socialisation and role overload to set forth specific hypotheses.

### 1.1 Role socialisation

The idea of ‘role socialisation’ is that smoking and drinking behaviours conflict with social expectations attached to responsible roles such as employee, partner or parent leading to reductions in these behaviours (Arnett, 2005, Chassin et al., 1992, Staff et al., 2010). Young people who make transitions into these responsible roles may therefore be less likely to smoke and drink heavily than those who participate in tertiary education. However, transitions into the role of employee should be distinguished from those into family roles such as partner or parent. Heightened expectations of responsibility after moving into employment may be mitigated by other similarities with the transition into tertiary education, such as smoking and drinking behaviours being an important part of social interaction in a new and unfamiliar environment (Pavis et al., 1998, Schulenberg and Maggs, 2002). On the other hand, transitions into family roles may amplify social control and leave less time for socialising with substance-using peers (Arnett, 2005, Staff et al., 2010). Thus, we hypothesise that early transitions into employment or family roles will both lead to less smoking and drinking in early adulthood than remaining in education, but that effects of family role transitions will be stronger than for transitions into work.

### 1.2 Role overload

‘Role overload’ is the notion that early transitions into adult roles can be overwhelming, creating stressful demands young people do not feel ready for (Burton, 2007, Schulenberg and Maggs, 2002). This overload may lead to smoking and drinking behaviours being used as coping mechanisms (Arnett, 2005, Kuntsche et al., 2005). While there may also be transitional stresses associated with participation in tertiary education, overload could be particularly likely if transitions are clustered together so that young people are dealing with many new things at the same time (Schulenberg and Maggs, 2002). Indeed for this reason it is important to take a holistic view of transitions, considering their spacing and timing. Where transitions into multiple roles occur very quickly over a short space of time, we might hypothesise that stress mechanisms would negate or over-take the benefits of transitions into responsible roles, leading to either comparable or higher levels of smoking and drinking than in tertiary education.

### 1.3 Estimating causal effects of transitions

It is important to acknowledge that transitional experiences do not occur randomly: they are associated with young people's background experiences. For example, those from more disadvantaged backgrounds, and those who already smoke or drink, have poorer mental health or are disengaged from school in adolescence, may be less likely to participate in tertiary education, and may make adulthood transitions at earlier ages (Cohen et al., 2003, Green et al., 2016, Sacker and Cable, 2010, Staff et al., 2010, Wickrama et al., 2010). Associations between smoking and drinking in early adulthood and transitional experiences may therefore be conflated with the influences of these background characteristics.

Our analyses use a propensity weighting procedure to adjust for background confounding and estimate causal effects of life transition patterns in comparison to tertiary education (Austin, 2011, Oakes and Johnson, 2006). The aim is to mimic a randomised experiment: we position participation in tertiary education *a priori* as a ‘control’ condition, and different patterns of life transitions as various alternative ‘exposures’. Weighting balances observed background characteristics across the exposure and control groups (whereas randomisation in an experiment balances both observed and unobserved characteristics across the groups). Specifically, we create a series of weights for those who participate in tertiary education so that, in terms of their background characteristics, they more closely resemble each of the different groups of people who do not participate. Such weighting enables estimation of the causal effect among the exposed (Austin, 2011), i.e. the effect that alternative patterns of life transitions had on those who experienced them, compared to if those same people had remained in education.

Propensity weighting does not remove the problem of unobserved confounding, so we may still question whether estimated effects are causal. Consistent evidence from different contexts strengthen the case for causality, whereas associations that are specific to particular populations are more likely to be spurious or local (Bradford Hill, 1965). Thus, we estimate effects of life transition patterns in three different cohorts. The 1958 National Child Development Study (NCDS58) and the 1970 British Birth Cohort Study (BCS70) both followed UK-wide cohorts over the transition to adulthood, separated by 12 years in time. The BCS70 cohort made their transition to adulthood during a rapid expansion of tertiary education (Bolton, 2012), and relative to NCDS58, a period in which smoking was less and alcohol consumption was more prevalent (British Beer and Pub Association, 2013, Office for National Statistics, 2016). We also examine the youth cohort of the West of Scotland: Twenty-07 Study (T07), who were making transitions to adulthood around the same time as the BCS70 but in the area in and around Glasgow: a large urban city, which underwent rapid deindustrialisation in the latter part of the Twentieth century, and had particularly poor employment prospects for those without tertiary education. Within these different contexts, both the background characteristics (observed or not) of those not in education, and the life transitions they made, may differ. If we can consistently observe the predicted patterns relating to the social roles inhabited and the timing and spacing of transitions, then the case for these as causal mechanisms is stronger.

### 1.4 Aims and hypotheses

Our main aims were to:

1) identify groups of young people with similar patterns of life transitions;
2) examine differences between the early adult smoking and drinking behaviours of those who made early transitions and those who participated in tertiary education;
3) compare these with estimates of the causal effects of each early transition pattern, using propensity weighting to create comparison groups who had remained in education but had similar background characteristics; and
4) see whether estimated effects were consistent across cohorts making the transition to adulthood in different contexts.

We hypothesised that, after adjusting for background confounding with propensity weighting, early transitions into responsible adult roles would be associated with less smoking and drinking in early adulthood than for those who participate in tertiary education, and that this difference would be quantifiably larger (i.e. with non-overlapping confidence intervals) for transitions into family roles such as partner or parent compared to transitions into employment only. For groups whose transitions are clustered closer together, we hypothesised that these differences would be weaker (again with non-over-lapping confidence intervals) or that the direction of association may even reverse. Finally, we hypothesised that the above patterns would be observed consistently (i.e. associations would be in the same direction and confidence intervals would overlap), despite contextual differences between cohorts.

## 2 Methods

### 2.1 Cohorts

The 1958 National Child Development Study (NCDS58) and the 1970 British Cohort Study (BCS70) both follow those born in the UK within particular weeks in 1958 and 1970 respectively (Plewis et al., 2004). The West of Scotland: Twenty-07 Youth Cohort (T07), born in 1971-2 and based in and around Glasgow, were followed from age 15 for 20 years (Benzeval et al., 2009). Analysis samples constituted those who participated in adolescent surveys and had data on the timing of at least one of their adulthood transitions (n = 11,230, n = 9364 and n = 1429 respectively for NCDS58, BCS70 and T07; representing 60.5%, 50.6% and 94.3% of the sample within each study). Data were mostly from surveys in adolescence and early adulthood, though retrospective data from later surveys was used to calculate transition timing. Adolescent surveys took place at age 16 in 1974 for NDS58, 1986 for BCS70, and 1987 for T07, and surveys in early adulthood took place in 1981 (at age 23) for NCDS58, in 1996 (at age 26) for BCS70, and 1993 (at age 22) for T07.

### 2.2 Measures

#### 2.2.1 Transition timing

Ages of key transitions (leaving full-time education, entering employment, entering a cohabiting relationship, having their first child, or leaving the parental home) were calculated from survey responses. In order to discount summer-time employment or education breaks, periods out of education or in employment were required to last at least five months before being counted as leaving or entering. Transition ages were categorised as follows: age 16 or before; ages 17–18; ages 19–21; or not by age 22 (focusing on transitions occurring before the age of 22, since early adult outcomes were observed between ages 22–26, depending on the cohort). Few respondents made early transitions into cohabitation and parenthood, so the first two categories were combined for entering a cohabiting relationship, and the variable for first child was collapsed into a binary indicator of whether or not this had occurred before age 22. In preliminary models, finer categorisations such as those adopted for the other transition measures yielded similar patterns; these broader categories were adopted in order to aid model convergence.

#### 2.2.2 Early adult outcomes

In early adulthood (ages 22–26, depending on study) respondents self-reported current smoking with one or more cigarettes per day coded as daily smoking. Respondents also reported their past week's drinking and numbers of alcohol units were derived (1 unit = 10 ml or 8 g of pure alcohol). Drinking more than 14 units for women or 21 units for men (Royal College of Physicians et al., 1995) was coded as heavy drinking in early adulthood.

#### 2.2.3 Background confounders

Gender, parental socioeconomic position (SEP), family structure, parental smoking and drinking, and adolescent smoking, drinking, psychiatric distress and school performance were all considered possible confounders in that they might influence the chances of both early adult smoking and drinking and of experiencing different transition patterns.

Parental SEP was measured using occupational class, income and education, with data from parents at adolescent surveys. Occupational class was coded according to the British Registrar General's classification (Office of Population Censuses and Surveys, 1980) as either non-manual (I, II and III non-manual) or manual (III manual, IV and V) using the highest status from couple parents. Weekly household income was equivalised for household composition to adjust for the balance of resources vs needs. Respondents were coded as low income if they were in the lowest tertile of equivalised income within each cohort, indicating disadvantage relative to other members of the cohort. A binary variable indicated whether at least one parent had remained in education beyond the age of 16 (i.e. indicating some parental engagement with tertiary education). Family structure indicated whether respondents were in single or two-parent families in adolescence.

Parents reported their smoking status when respondents were aged 16, and respondents in BCS70 also reported on their parents' smoking status. Any indication (parental or adolescent report) of either parent smoking was coded as parental smoking. Measures of parental drinking differed considerably: in NCDS58 the only available indicator was an interviewer assessment (at age 7) of whether or not the family was having difficulties with alcoholism; in BCS70 at age 16, respondents and parents reported on parental drinking and respondent reports of either parent drinking alcohol ‘most days’ (as opposed to never, occasionally or some days), or parental reports of drinking alcohol ‘3 or 4 times a week’ or more were coded as heavy parental drinking. T07 parents reported alcohol consumed over the past week during adolescent surveys. Either parent consuming more than 14 units for women or 21 units for men (Royal College of Physicians et al., 1995) was coded as heavy parental drinking.

Respondents self-reported smoking and drinking behaviours in adolescence. Daily adolescent smoking was coded using the closest available definition (10 or more cigarettes weekly for NCDS58; 6 or more weekly for BCS70; and 7 or more weekly for T07). Weekly drinking was the most consistent measure of adolescent drinking available, based either on questions about regular drinking frequency, or on past week consumption if this was unavailable. The 12-item General Health Questionnaire (GHQ-12) was administered in adolescence in BCS70 and T07, and scores of 3 or more were used to indicate psychiatric distress (Banks, 1983). In NCDS58 the neuroticism component of the Rutter behavioural scale (four items with face validity for psychiatric distress, each scored 0–2; Rutter, 1967) as rated by the young person's school teacher at age 16 was employed with scores of 2 or more indicating distress. The most consistent measure of adolescent school performance available in all three cohorts was a binary indicator of whether they were taking 7 or more nationally certificated exams in school that year.

### 2.3 Analyses

Analyses were stratified by cohort and performed in Mplus 7 (Muthén and Muthén, 1998-2012) and SPSS (v21). Respondents were first classified into groups via latent class analysis (Collins and Lanza, 2010) of the five transition timing variables. Although models were constructed within each cohort separately, solutions with the same number of classes in each cohort were preferred *a priori*, to facilitate cross-cohort comparison. Classes were added iteratively until models failed to converge, had difficulties replicating solutions, were clearly fitting less well with additional classes, or additional classes represented very small sub-groups (less than 5% of the sample). Gender differences were not statistically significant in T07 and produced substantively similar classes in the other two cohorts, so latent classes were constrained to be gender-invariant, whilst allowing class membership to vary by gender. Respondents were then assigned to the class for which they had the highest probability of membership.

Having established a classification of transition timing, propensity weighting (Austin, 2011) was used to adjust for background confounding of associations between transition timing and smoking and drinking in early adulthood. This differs from propensity matching procedures in that propensity scores are used to weight members of a control group so that they more closely resemble each comparison group, rather than to match specific participants with similar characteristics. Since propensity weighting requires complete data on all background confounders, missing values were estimated using multiple imputation (25 imputations; Asparouhov and Muthén, 2010). The imputation model was unconstrained, including all the background confounders, the early adult outcomes, and variables indicating the probability of being in each early transition class (estimated individually for each respondent from their observed responses using the latent class model).

Within imputed datasets, propensity scores were derived from multinomial logistic regression (in SPSS v21) of transition class on background confounders (with interactions between gender and each other predictor). Propensity scores for each respondent represent their probability of (or propensity for) being in each class, given their background characteristics. Propensity scores were used to calculate four sets of analysis weights, each comparing one early transition class to the tertiary education class. For each set of weights, those in the early transition class were assigned a weight of 1, whilst weights for those in tertiary education were calculated by dividing their propensity for being in the early transition class by their propensity for being in the tertiary education class (while those in other early transition classes were assigned a weight of 0 to exclude them from the comparison). This gives extra weight to participants in tertiary education who have similar background characteristics to those in the early transition group, and less weight to those who have different background characteristics.

In order to check how well weighting balanced background characteristics, we examined standardised mean differences between those in early transition classes and those in tertiary education on all background confounders. Standardised mean differences less than 0.2 were taken to indicate sufficient balance had been achieved (Lanza et al., 2013) and that the weighting model did not require further revision. We also checked covariate balance with weights truncated at their 95th percentile (Cole and Hernán, 2008), to see whether balance was only being achieved by relying heavily on outliers.

The final step was estimating the effects of transition class membership on early adult smoking and drinking, using a logistic regression of each outcome separately on a binary indication of transition class (early transition class vs. tertiary education). This was carried out both with and without the propensity weights, in order to ascertain how much of the overall association could be accounted for by background confounding.

## 3 Results

### 3.1 Missing data and descriptive statistics

Supplementary Table 1 shows proportions of missing data for variables in each cohort. The highest levels of missing data were among background confounders in BCS70 (the age 16 survey was split across multiple questionnaires, some of which were administered through schools and response rates were hampered by teacher strikes, though this had little impact on sample characteristics; Shepherd, 1997). Supplementary Table 2 shows descriptive statistics. Early adult smoking rates were lower in BCS70 and T07 than in NCDS58. Heavy drinking in early adulthood declined somewhat from NCDS58 to BCS70 but was highest in T07.

### 3.2 Latent class analysis

Fit statistics (log-likelihood, AIC, BIC) for NCDS58 and BCS70 continued to improve up to 8 classes when additional classes became small, representing less than 5% of the population (see Supplementary Table 3). In T07 the BIC favoured a five-class solution, whilst the AIC favoured seven classes, and the log-likelihood continued to improve with additional classes. Five class solutions for all cohorts were examined, and were similar in interpretation for BCS70 and T07, but differed for one class in NCDS58. A six class model was adopted as it resolved this discrepancy. There were statistically significant differences in the six-class pattern between cohorts (chi-square test for constraining parameters equal across cohorts: p < 0.001) but this may have been due to the large sample size making this a sensitive test, as the six classes had similar interpretations in each cohort.

**Fig. 1:**
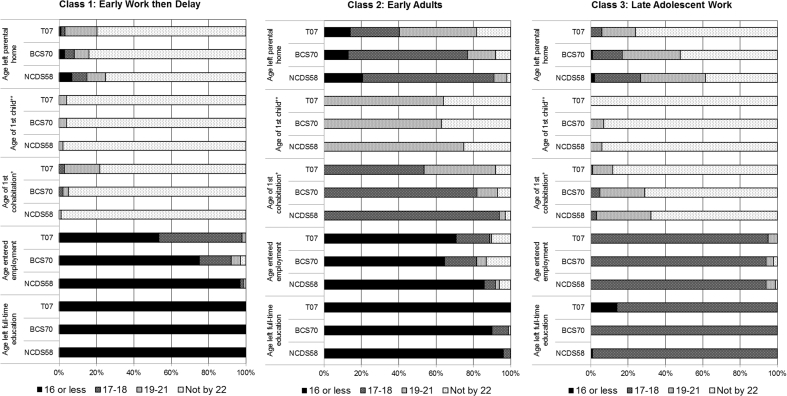

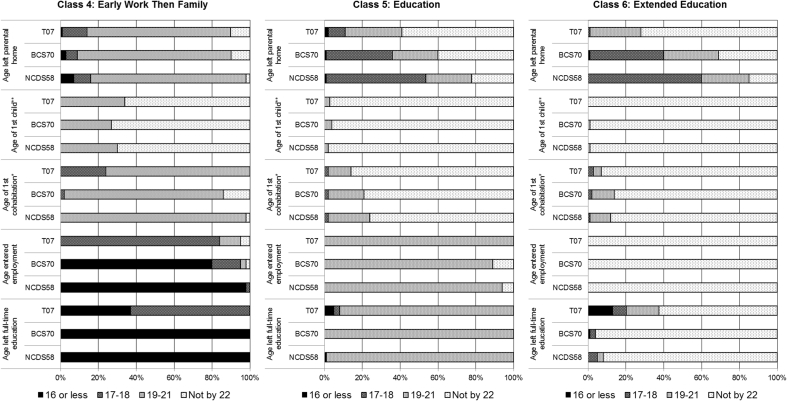
Description of transitional classes in each cohort. *For age of 1st cohabitation 17–18 means 18 or less. **For age of 1st child 19–21 means 21 or less.

**Fig. 2:**
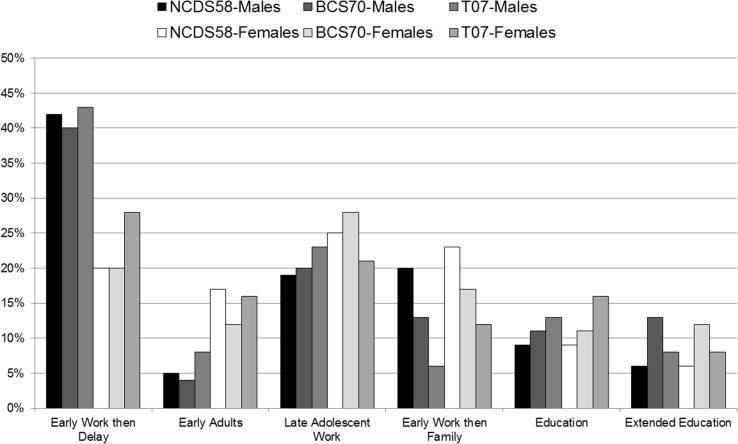
Estimated prevalence of latent classes by gender and cohort.

Fig. 1 shows response probabilities for each latent class in each cohort and Fig. 2 shows the estimated prevalence of each latent class by gender and cohort. The first latent class, *Early Work then Delay*, comprised those who left school at age 16 or earlier, entered employment (almost) immediately, but then delayed other transitions. For males, this was the largest group in all cohorts with an estimated prevalence of 40–43%. Prevalence was lower for females but still high, including an estimated 20% from NCDS58 and BCS70 and 28% from T07.

The next class, labelled *Early Adults*, made all five transitions at relatively early ages. This was one of the smallest groups for males with an estimated prevalence of 5–8%, whilst for females this group accounted for 16–17% in NCDS58 and T07 and 12% in BCS70.

The third class was labelled *Late Adolescent Work*; they tended to leave school and enter employment between 17 and 18 years of age. Other transitions had generally not occurred by age 22 in this group. This group represented 19–28% of the males and females in each cohort.

The fourth class was labelled *Early Work then Family*; they tended to leave home and start cohabiting between the ages of 19–21 after early transitions out of school and into work. They were also more likely to begin parenthood by age 22 than any other group besides the *Early Adults*. The *Early Work then Family* pattern was most prevalent in NCDS58 (20% for males and 23% for females), with a decrease in prevalence among the more recent cohorts, especially for males, dropping as low as an estimated 6% for T07 males.

A fifth group, labelled *Education* had not begun cohabiting or child-bearing by age 22, and did not make transitions from education to employment until ages 19–21. In NCDS58 approximately half of the respondents in this group had left home by ages 17–18. Respondents from this group in BCS70 were more evenly balanced across age categories for leaving home. In T07 most respondents in this group had not left home by age 22.

The sixth class, labelled *Extended Education*, exhibited a very similar pattern of transitions to those in the *Education* class except that most had not yet transitioned from education into employment by age 22. The patterning of leaving home by cohort was similar, with those in T07 least likely to have left home by age 22.

Thus, patterns of early transitions (*Early Work then Delay, Early Adults, Late Adolescent Work, and Early Work then Family*) and educational participation (*Education, Extended Education*) were identified. Combining across the *Education* and *Extended Education* patterns, respondents in NCDS58 were less likely to delay transitions to remain in education than those in the more recent cohorts. Between the two more recent cohorts, those from T07 tended more towards the *Education* pattern and those from BCS70 towards the *Extended Education* pattern. Since these last two classes both represented a pattern of delaying transitions to remain in education, they were combined for subsequent analysis into one group labelled *Tertiary Education*, which was used as the reference group against which to compare the other early transition groups. Preliminary analyses suggested similar associations with background factors for these two groups, and combining them resulted in a larger comparison group, helping to avoid difficulties associated with small numbers.

### 3.3 Performance of propensity weights

**Fig. 3:**
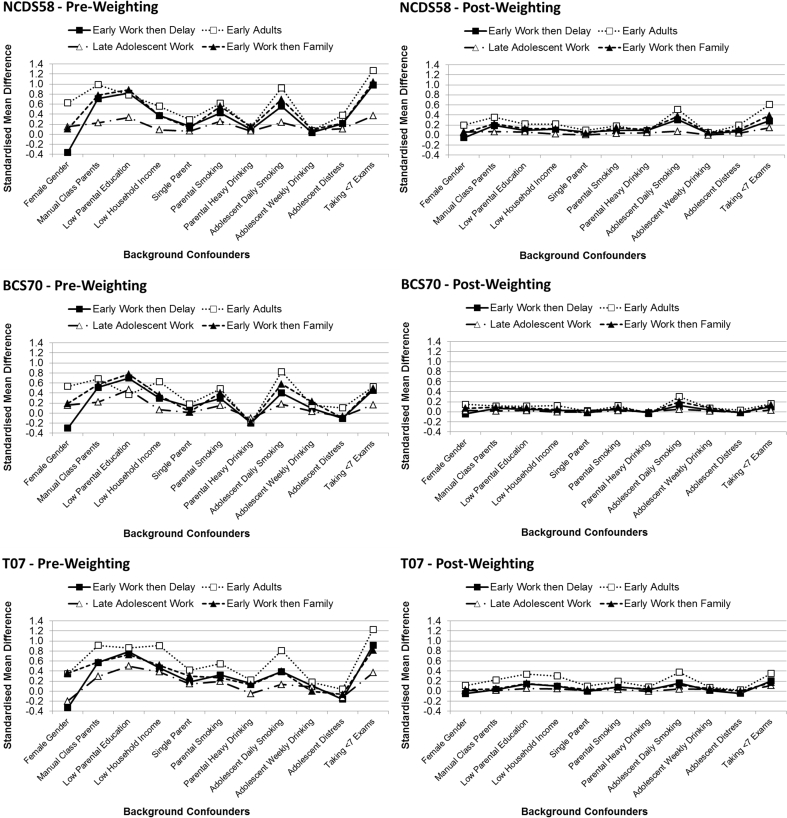
Standardised Mean Differences on Background Confounders (comparing to the *Tertiary Education* group).

Variability in the weights was high, which can lead to imprecision in estimates (Cole and Hernán, 2008) so we present analyses with weights for the *Tertiary Education* group truncated at their 95th percentile. A parallel analysis without truncation of the weights is presented in Supplementary File 4 and differences in findings are detailed below. Standardised mean differences between each of the early transition groups and the *Tertiary Education* group are shown for background confounders in Fig. 3 (averaging across imputed data-sets). Prior to weighting there were substantial between group differences on these factors, following a roughly similar (but not identical) pattern across the three cohorts, with the largest differences in gender, background SEP, parental and adolescent smoking, and school performance. Fig. 3 shows that the truncated weights reduced the differences between the early transition groups and the *Tertiary Education* group for most background factors to fall within the pre-defined acceptable range (−0.2 to 0.2; Lanza et al., 2013). Thus, the truncated propensity weights largely accounted for selection biases on the basis of these background confounders, meaning we can be more confident that associations estimated with the weighting represent causal effects. However, while covariate balance was achieved with the full weights (see supplementary file 4), the truncated weights resulted in some residual imbalance. *Early Adults* were not fully balanced on adolescent smoking in any cohort, or on SEP in NCDS58 and T07. Additionally, in NCDS58 the *Early Work then Delay* and *Early Work then Family* groups retained some imbalance on adolescent smoking and school performance. This indicates that these groups in these cohorts were so different from those in *Tertiary Education* that balance on these characteristics could not be achieved without relying heavily on a small number of cases. There may also be unobserved background factors, which lead to differences between the groups which we are unable to account for. Thus, the results should be viewed as *estimates* of causal effects, based on an assumption of no further unmeasured confounding, and estimates for *Early Adults* (in all cohorts) and the *Early Work then Delay* and *Early Work then Family* groups in NCDS58 should be treated with particular caution as we were not able to fully remove observed background confounding.

### 3.4 Propensity weighted estimation of effects

We then compared the early adult smoking and drinking of each early transition group to those in tertiary education. We did this with and without the weighting, in order to understand how much of the overall associations might represent a causal effect. Table 1 shows estimated associations for early adult smoking in each cohort. Most of the early transition groups were associated with higher odds of smoking in early adulthood than in *Tertiary Education*. ORs were highest for *Early Adults* and the only exception was the *Late Adolescent Work* group in T07. Applying the weights accounted for substantial portions of these associations (ranging from 22 to 69%), but potentially causal contributions of early transitions to early adult smoking were present for most groups. After weighting the confidence intervals for *Early Adults* over-lapped with those of other groups, and associations were generally larger in NCDS58 than in BCS70. Use of the full weights resulted in greater attenuation of the effects and wider confidence intervals, such that only the NCDS58 differences were replicated, and then not for the *Early Adults* (see Supplementary File 4).

**Table 1:** ORs for early adult smoking.

	Pre-Weighting			Post-Weighting			% difference
	OR	95% CI	P-value	OR	95% CI	P-value	
*Early Work then Delay (ref: Tertiary education)*							
NCDS58	2.78	2.43–3.18	<0.001	2.35	1.88–2.95	<0.001	−24
BCS70	1.94	1.68–2.25	<0.001	1.41	1.18–1.69	<0.001	−56
T07	1.76	1.26–2.46	0.001	1.24	0.78–1.98	0.370	−69
*Early Adult (ref: Tertiary education)*							
NCDS58	4.91	4.16–5.79	<0.001	3.59	2.73–4.74	<0.001	−34
BCS70	3.31	2.67–4.11	<0.001	2.02	1.56–2.62	<0.001	−56
T07	4.72	2.96–7.53	<0.001	3.72	1.94–7.13	<0.001	−27
*Late Adolescent Work (ref: Tertiary education)*							
NCDS58	1.66	1.45–1.90	<0.001	1.45	1.24–1.71	<0.001	−31
BCS70	1.31	1.13–1.52	<0.001	1.16	1.00–1.35	0.053	−48
T07	0.90	0.60–1.34	0.599	0.79	0.51–1.25	0.315	+101
*Early Work then Family (ref: Tertiary education)*							
NCDS58	3.25	2.81–3.75	<0.001	2.57	2.04–3.25	<0.001	−30
BCS70	2.33	1.93–2.82	<0.001	1.58	1.28–1.95	<0.001	−57
T07	1.84	1.18–2.88	0.007	1.84	0.94–2.91	0.079	−22

Table 2 shows weighted and unweighted ORs for heavy drinking in early adulthood. In unweighted analyses in NCDS58 and BCS70 the *Early Adult, Late Adolescent Work* and *Early Work then Family* groups were associated with reduced odds of heavy drinking compared to those in *Tertiary Education.* These associations all remained after weighting but were attenuated by 13–32% (those for NCDS58 were not replicated with the full weights; see supplementary file 4). The *Early Work then Delay* group showed a different pattern with a trend towards reduced odds in BCS70, but clearly raised odds of heavy drinking in NCDS58 and a similar borderline trend in T07, before and after weighting. In T07 none of the other associations were significant regardless of weighting.

**Table 2:** ORs for early adult heavy drinking.

	Pre-Weighting			Post-Weighting			% difference
	OR	95% CI	P-value	OR	95% CI	P-value	
*Early Work then Delay (ref: Tertiary education)*							
NCDS58	1.47	1.29–1.67	<0.001	1.40	1.14–1.74	0.002	−14
BCS70	0.93	0.81–1.07	0.330	0.93	0.77–1.12	0.462	0
T07	1.35	0.99–1.84	0.061	1.56	0.98–2.49	0.061	+61
*Early Adult (ref: Tertiary education)*							
NCDS58	0.46	0.38–0.56	<0.001	0.61	0.45–0.83	0.002	−28
BCS70	0.41	0.30–0.55	<0.001	0.52	0.36–0.74	<0.001	−19
T07	0.67	0.41–1.10	0.112	1.33	0.68–2.61	0.405	−201
*Late Adolescent Work (ref: Tertiary education)*							
NCDS58	0.85	0.73–0.98	0.022	0.87	0.74–1.01	0.070	−13
BCS70	0.70	0.60–0.82	<0.001	0.79	0.67–0.95	0.010	−32
T07	1.13	0.78–1.64	0.502	1.16	0.77–1.76	0.474	+21
*Early Work then Family (ref: Tertiary education)*							
NCDS58	0.65	0.56–0.75	<0.001	0.70	0.55–0.88	0.002	−14
BCS70	0.53	0.43–0.65	<0.001	0.60	0.46–0.79	<0.001	−16
T07	0.74	0.47–1.17	0.197	1.39	0.79–2.46	0.255	−252

## 4 Discussion

### 4.1 Principal findings

Five groupings relating to the timing of adulthood transitions were identified. Four groups making early transitions (*Early Work then Delay, Early Adults, Late Adolescent Work,* and *Early Work then Family*) were compared to a *Tertiary Education* group who remained in education, delaying transitions into work and family roles. Early transitions tended (with some exceptions) to be associated with higher levels of early adult smoking but lower levels of drinking than in the *Tertiary Education* group. Most of these associations were attenuated when adjusting for background confounding using propensity weighting, but significant associations remained. There was little support for role socialisation for smoking; most associations were not in the expected direction. There was some tentative support for role overload as the *Early Adult* group, who had the most clustered pattern of transitions, had the highest risk for smoking, but the confidence intervals of these associations did not clearly separate them from the estimates for other transition groups.

There was partial support for socialisation effects on heavy drinking in BCS70 and NCDS58 with the *Early Adult, Late Adolescent Work* and *Early Work then Family* groups all having reduced odds relative to those in *Tertiary Education*. Associations were stronger in magnitude for the family transitions groups (*Early Adults, Early Work then Family*) than the *Late Adolescent Work* group (who rarely had family transitions), but not unambiguously so as confidence intervals overlapped. The *Early Adult* group (most clustered transitions) showed similar associations to the other early transition groups, so there was little support for role overload effects on drinking. However, increased odds of heavy drinking for the *Early Work then Delay* group were seen in two cohorts (NCDS58 and T07), which is consistent with the hypothesis that socialisation effects would be less evident where transitions were into employment but not family roles.

### 4.2 Strengths and weaknesses

Interpretation of the propensity weighted effects as causal assumes no confounding (measured or unmeasured). While several potentially important confounders were included, there were strong differences in some background characteristics. This observed confounding was not entirely removed by the weighting, so some transition groups differed on important factors. Particularly, findings for *Early Adults* remained confounded (though to a lesser degree than in unadjusted analyses) by adolescent smoking and school performance in all cohorts (and by SEP in NCDS58 and T07), as did the *Early Work then Delay* and *Early Work then Family* groups in NCDS58. Thus it remains unclear how much of the estimated associations for these groups represent confounding rather than causal mechanisms. Residual confounding from other factors (such as parental mental health or adolescent time-discounting, where more distant rewards are valued less) also remains possible as measures of these were not available in all cohorts. However, these may be accounted for to some extent if they were correlated with factors that were included and were balanced across groups. Further, we focused on timing of first transitions into adult roles, without addressing the success with which transitions were negotiated, or maintenance of roles beyond first transitions, though those who make transitions earliest may be least likely to manage them successfully (Chassin et al., 1992, Furlong et al., 2003).

It is important to emphasise that our findings come from historical cohorts, making transitions to adulthood in the 1980s and 1990s, and may not generalise to transitional experiences in the UK today. The role transition patterns observed here may differ from those in recent years as demographic trends towards increasing educational participation and delaying of partnering and parenthood have continued (Clarke and Roberts, 2011, Machin and Vignoles, 2004) alongside an increasing trend for adult children to co-reside with their parents (Berrington et al., 2014). Nevertheless, the effects of social role transitions on substance use in early adulthood were stable over the 1990s and 2000s in the US (Staff et al., 2010), and associations between tertiary education and alcohol use have been observed in various other US studies (Carter et al., 2010) as well as in New Zealand (Kypri et al., 2005), so the associations may be generalisable.

No significant differences were observed in T07 after propensity weighting. This may represent lack of statistical power, this being the smallest of the three cohorts, but many trends also differed in direction to those in the other cohorts. The absence of or different direction of associations may reflect qualitative differences in the *Tertiary Education* experience in this cohort compared to the others, either remaining longer in the parental home or the larger role played by further education colleges in delivering tertiary education in Scotland (Iannelli, 2007).

### 4.3 Comparison with other studies

Support for role socialisation effects on alcohol but not cigarette use is consistent with some prior US research (Bachman et al., 1984). However, in a more recent study, which analysed within-person change to control for confounding from stable individual differences, while drinking was more strongly influenced than cigarette use, both reduced with family role transitions (Staff et al., 2010). We did not examine the mechanisms of early transition effects directly, but they found reductions in time spent socialising with peers, and increases in religiosity were key mediators of family role socialisation effects (Staff et al., 2010). In another US study which classified young people according to their life transitions those making family transitions were less likely to have trajectories of increasingly frequent drinking and of increasing cigarette use during young adulthood (Huh et al., 2013). Together, these studies support our findings in relation to drinking, but differ on smoking, which could be explained by residual background confounding in our study, or differences in study context (see below).

### 4.4 Meaning and implications

Findings supported role socialisation in relation to early adult heavy drinking (but not smoking), and tentatively supported role overload effects in relation to early adult smoking (but not drinking). They suggest that if those in the early transition groups had instead delayed their transitions to remain in education, they may have experienced protection against smoking, but increased risk for heavy drinking. While heavy drinking among students may be transitory, returning to safer levels in time (Schulenberg and Maggs, 2002), more frequent drinking has also been found among highly educated members of NCDS58 and BCS70 at ages 30–33 (Schoon et al., 2012). Even if habits do not persist into later life, there might be immediate consequences for the individual and those around them. Alcohol is often involved in the most common types of mortality among young people: accidents, homicides and suicides (Mason et al., 2008); and many people experience harms from other people's drinking (Gell et al., 2015). Some experts therefore rate alcohol as substantially more harmful than tobacco (Nutt et al., 2010).

Role socialisation effects on smoking may be less evident because smoking is more addictive than alcohol (Chassin et al., 1992, Nutt et al., 2010), and thereby less susceptible to social control. Another explanation may be that smoking conflicts less than heavy drinking with the social expectations attached to work and family roles. This may seem counter-intuitive; health risks of smoking are now well understood (Jarvis and Wardle, 2006), while even recent research suggests heavy drinking may be seen as normative and unproblematic (Lovatt et al., 2015). Nevertheless, the adverse impacts of smoking are mainly limited to its long-term health risks, while drinking can have more immediate impacts on role performance via impaired decision making and functioning (e.g. hang-overs etc.). Weaker effects for smoking may indicate long-term health risks are not very salient in the expectations of responsibility attached to work and family roles, some even suggest discounting such risks is a feature of this stage of life (the “age of possibilities”; Arnett, 2005). Indeed, while drinkers may disregard health risks, they do cite fulfilment of work and family responsibilities as reasons for moderation of drinking (Lovatt et al., 2015).

The increased risks for smoking observed in early transition groups could be interpreted as an indication of role overload, especially since the largest associations were consistently found among the *Early Adults*. The stress of taking on new roles may increase motivations to self-medicate with substance use (Arnett, 2005, Schulenberg and Maggs, 2002), but if family roles restrict opportunities for social drinking (Staff et al., 2010), perhaps young people turn to cigarettes, which are less socially oriented (Pearson et al., 2006) and more addictive (Nutt et al., 2010) than alcohol, as an alternative form of self-medication.

### 4.5 Future research

Findings showed heterogeneity across cohorts, and conflicted in some instances with US data (see above), so if early role transitions do have causal effects, these may be moderated by or dependent upon contextual conditions. Comparing our two national cohorts, the strongest evidence we found for early role transition effects were in those cohorts where population consumption levels were higher (i.e. NCDS58 for smoking, and BCS70 for drinking). Perhaps mechanisms are weaker when behaviours are less normative: one explanation for the discrepancy with recent US data on smoking could be that reductions in the social acceptability of smoking have weakened tendencies to self-medicate with cigarettes, bringing socialisation effects to the fore. We compared across cohorts to see if effects were consistent across different patterns of early transitions with different confounding structures. However, early transition patterns and background confounding were more similar than different across these cohorts. Further research across varied situations and confounding structures could help identify the conditions under which role socialisation and overload mechanisms are causal. For example, a study on Hispanics in Southern California found that starting a job or a romantic partnership were both associated with increased odds of cigarette use and binge drinking after a similar adjustment for background confounding to that applied here (Allem et al., 2016), contrasting with other findings from the US. A wider evidence base on transition effects within different populations and contexts could help in discerning consistent patterns.

### 4.6 Conclusions

Varied patterns of transitions into work and family roles were compared with remaining in education. Causal effect estimates for these transitions (based on propensity weighting) suggested that wider participation in tertiary education could have health benefits in reduced risk for smoking, but also some adverse impacts on drinking behaviour, via delay of adult role transitions. Continuing to widen access to tertiary education, with its health and other benefits, whilst intervening to reduce adverse effects on drinking, seems sensible. For example, institutions of tertiary education may want to intervene to ameliorate norms of heavy drinking among students, help students manage transitions, or offer support for those with heavy drinking habits. Policy-makers might aim to encourage or support such measures.

## References

[bib1] Allem J.-P., Sussman S., Soto D.W., Baezconde-Garbanati L., Unger J.B. (2016). Role transitions and substance use among Hispanic emerging adults: a longitudinal study using coarsened exact matching. Addict. Behav..

[bib2] Arnett J.J. (2000). Emerging adulthood: a theory of development from the late teens through the twenties. Am. Psychol..

[bib3] Arnett J.J. (2005). The developmental context of substance use in emerging adulthood. J. Drug Issues.

[bib4] Asparouhov T., Muthén B.O. (2010). Multiple Imputation with Mplus.

[bib5] Austin P.C. (2011). An introduction to propensity score methods for reducing the effects of confounding in observational studies. Multivar. Behav. Res..

[bib6] Bachman J.G., O'Malley P.M., Johnston L.D. (1984). Drug use among young adults: the impacts of role status and social environment. J. Personality Soc. Psychol..

[bib7] Banks M.H. (1983). Validation of the general health questionnaire in a young community sample. Psychol. Med..

[bib48] Benzeval M., Der G., Ellaway A., Hunt K., Sweeting H., West P. (2009). Cohort profile: west of Scotland twenty-07 study - health in the community. Int. J. Epidemiol..

[bib8] Berrington A., Tammes P., Roberts S. (2014). Economic Precariousness and Living in the Parental Home in the UK. http://www.cpc.ac.uk/publications/cpc_working_papers/pdf/2014_WP55_Economic_precariousness_and_living_in_the_parental_home_in_the_UK_Berrington_et_al.pdf.

[bib9] Bolton P. (2012). Education: Historical Statistics. Commons Briefing Papers SN04252. http://researchbriefings.parliament.uk/ResearchBriefing/Summary/SN04252.

[bib10] Bradford Hill A. (1965). The environment and disease: association or causation?. Occup. Med..

[bib11] British Beer, Pub Association (2013). UK alcohol consumption 1900-2013. Ind. Briefings.

[bib12] Burton L. (2007). Childhood adultification in economically disadvantaged families: a conceptual model. Fam. Relat..

[bib13] Carter A., Brandon K., Goldman M. (2010). The college and noncollege experience: a review of the factors that influence drinking behaviour in young adulthood. J. Stud. Alcohol Drugs.

[bib14] Chassin L., Presson C.C., Sherman S.J., Edwards D.A. (1992). The natural history of cigarette smoking and young adult social roles. J. Health Soc. Behav..

[bib15] Clarke L., Roberts C., Wadsworth M., Bynner J. (2011). Family structure and family policy and practice. A Companion to Life Course Studies: the Social and Historical Context of the British Birth Cohort Studies.

[bib16] Cohen P., Kasen S., Chen H., Hartmark C., Gordon K. (2003). Variations in patterns of developmental transitions in the emerging adulthood period. Dev. Psychol..

[bib17] Cole S.R., Hernán M.A. (2008). Constructing inverse probability weights for marginal structural models. Am. J. Epidemiol..

[bib18] Collins L.M., Lanza S.T. (2010). Latent Class and Latent Transition Analysis with Applications in the Social, Behavioral, and Health Sciences.

[bib19] Côté J., Bynner J. (2008). Changes in the transition to adulthood in the UK and Canada: the role of structure and agency in emerging adulthood. J. Youth Stud..

[bib20] Furlong A., Cartmel F., Biggart A., Sweeting H., West P. (2003). Youth Transitions: Patterns of Vulnerability and Processes of Social Inclusion.

[bib21] Gell L., Ally A., Buykx P., Hope A., Meier P.S. (2015). Alcohol's Harm to Others.

[bib22] Graham H. (2007). Unequal Lives: Health and Socioeconomic Inequalities.

[bib23] Green M.J., Leyland A.H., Sweeting H., Benzeval M. (2016). Adolescent smoking and tertiary education: opposing pathways linking socio-economic background to alcohol consumption. Addiction.

[bib24] Huh J., Huang Z., Liao Y., Pentz M.A., Chou C.P. (2013). Transitional life events and trajectories of cigarette and alcohol use during emerging adulthood: latent class analysis and growth mixture modeling. J. Stud. Alcohol Drugs.

[bib25] Iannelli C. (2007). Inequalities in entry to higher education: a comparison over time between Scotland and England and Wales. High. Educ. Q..

[bib26] Jarvis M.J., Wardle J., Marmot M., Wilkinson R.G. (2006). Social patterning of individual health behaviours: the case of cigarette smoking. Social Determinants of Health.

[bib27] Kuntsche E., Knibbe R., Gmel G., Engels R. (2005). Why do young people drink? A review of drinking motives. Clin. Psychol. Rev..

[bib28] Kypri K., Cronin M., Wright C.S. (2005). Do university students drink more hazardously than their non-student peers?. Addiction.

[bib29] Lanza S.T., Coffman D.L., Xu S. (2013). Causal inference in latent class analysis. Struct. Equ. Model..

[bib30] Lovatt M., Eadie D., Meier P.S., Li J., Bauld L., Hastings G. (2015). Lay epidemiology and the interpretation of low risk drinking guidelines by adults in the United Kingdom. Addiction.

[bib31] Machin S., Vignoles A. (2004). Educational inequality: the widening socio-economic gap. Fisc. Stud..

[bib32] Mason A.W., Kosterman R., Haggerty K.P., Hawkins J.D., Redmond C., Spoth R.L. (2008). Dimensions of adolescent alcohol involvement as predictors of young-adult major depression. J. Stud. Alcohol Drug Use.

[bib33] Muthén L.K., Muthén B.O. (1998-2012). Mplus User's Guide.

[bib34] Nutt D.J., King L.A., Phillips L.D. (2010). Drug harms in the UK: a multicriteria decision analysis. Lancet.

[bib35] Oakes J.M., Johnson P.J., Oakes J.M., Kaufman J.S. (2006). Propensity score matching for social epidemiology. Methods in Social Epidemiology.

[bib36] Office for National Statistics (2016). Adult Smoking Habits in Great Britain. https://www.ons.gov.uk/peoplepopulationandcommunity/healthandsocialcare/drugusealcoholandsmoking/datasets/adultsmokinghabitsingreatbritain.

[bib49] Office of Population Censuses and Surveys (1980). Classification of Occupations.

[bib37] Pavis S., Cunningham-Burley S., Amos A. (1998). Health-related behavioural change in context: young people in transition. Soc. Sci. Med..

[bib38] Pearson M., Sweeting H., West P., Young R., Gordon J., Turner K. (2006). Adolescent substance use in different social and peer contexts: a social network analysis. Drugs Educ. Prev. policy.

[bib39] Plewis I., Calderwood L., Hawkes D., Nathan G. (2004). National Child Development Study and 1970 British Cohort Study: Technical Report: Changes in the NCDS and BCS70 Populations and Samples over Time.

[bib40] Royal College of Physicians, Royal College of Psychiatrists, Royal College of General Practitioners (1995). Alcohol and the Heart in Perspective: Sensible Limits Reaffirmed.

[bib41] Rutter M. (1967). A Children's behaviour questionnaire for completion by teachers: preliminary findings. J. Child Psychol. Psychiatry.

[bib42] Sacker A., Cable N. (2010). Transitions to adulthood and psychological distress in young adults born 12 years apart: constraints on and resources for development. Psychol. Med..

[bib43] Schoon I., Chen M., Kneale D., Jager J. (2012). Becoming adults in Britain: lifestyles and wellbeing in times of social change. Longitud. Life Course Stud..

[bib44] Schulenberg J.E., Maggs J.L. (2002). A developmental perspective on alcohol use and heavy drinking during adolescence and the transition to young adulthood. J. Stud. Alcohol.

[bib45] Shepherd P., Bynner J., Ferrie E., Shepherd P. (1997). Survey and response. Getting on, Getting by, Getting Nowhere. Twenty-somethings in the 1990's.

[bib46] Staff J., Schulenberg J.E., Maslowsky J., Bachman J.G., O'Malley P.M., Maggs J.L. (2010). Substance use changes and social role transitions: proximal developmental effects on ongoing trajectories from late adolescence through early adulthood. Dev. Psychopathol..

[bib47] Wickrama T., Wickrama K.A.S., Baltimore D.L. (2010). Adolescent precocious development and young adult health outcomes. Adv. Life Course Res..

