# Supplementary material for: Causal effects of transitions to adult roles on early adult smoking and drinking: Evidence from three cohorts
Source: Soc Sci Med. 2017 Aug;187:193–202. doi: 10.1016/j.socscimed.2017.06.018 (PMC5529289; doi:10.1016/j.socscimed.2017.06.018)
Supplement: Table S1 [file mmc1.docx]

**Supplementary Table 1: Proportions with missing data for analysis variables**

|  | **NCDS58** |  | **BCS70** |  | **T07** |  |
| --- | --- | --- | --- | --- | --- | --- |
|  | N | % | N | % | N | % |
|  | | | | | | |
| *Transition timing* | | | | | | |
| **Leaving Education** | 0 | 0.0 | 28 | 0.3 | 119 | 8.3 |
| **Entering Employment** | 173 | 1.5 | 112 | 1.2 | 221 | 15.5 |
| **Entering Cohabitation** | 35 | 0.3 | 504 | 5.4 | 512 | 35.8^a^ |
| **First Child** | 1 | 0.0 | 775 | 8.3 | 292 | 20.4 |
| **Leaving Parental Home** | 141 | 1.3 | 1,212 | 12.9 | 168 | 11.8 |
| *Early Adult Outcomes* |  |  |  |  |  |  |
| **Daily Smoking** | 39 | 0.3 | 3,092 | 33.0 | 268 | 18.8 |
| **Heavy Drinking** | 8 | 0.1 | 2,934 | 31.3 | 273 | 19.1 |
| *Background Confounders* | | | | | | |
| **Gender** | 0 | 0.0 | 0 | 0.0 | 0 | 0.0 |
| **Parental Occupational Class** | 2,187 | 19.5 | 3,443 | 36.8 | 20 | 1.4 |
| **Parental Education** | 1,940 | 17.3 | 3,259 | 34.8 | 22 | 1.5 |
| **Household Income** | 3,764 | 33.5 | 4,096 | 43.7 | 85 | 5.9 |
| **Family Structure** | 1,822 | 16.2 | 4,649 | 49.6 | 34 | 2.4 |
| **Parental Smoking** | 1,874 | 16.7 | 1,037 | 11.1 | 107 | 7.5 |
| **Parental Drinking** | 2,658 | 23.7 | 1,237 | 13.2 | 116 | 8.1 |
| **Adolescent Smoking** | 1,722 | 15.3 | 3,741 | 40.0 | 6 | 0.4 |
| **Adolescent Drinking** | 1,697 | 15.1 | 3,801 | 40.6 | 3 | 0.2 |
| **Adolescent Distress** | 1,570 | 14.0 | 5,080 | 54.3 | 84 | 5.9 |
| **School Performance** | 1,610 | 14.3 | 6,283 | 67.1 | 10 | 0.7 |
|  |  |  |  |  |  |  |
| **Denominator** | 11,230 | - | 9,364 | - | 1,429 | - |
|  |  |  |  |  |  |  |

^a^There was a particularly high level of missing data for cohabitation timing in this cohort, primarily due to it being ascertained retrospectively at age 35 after a portion of the sample had dropped out of the study.
