# Supplementary material for: Causal effects of transitions to adult roles on early adult smoking and drinking: Evidence from three cohorts
Source: Soc Sci Med. 2017 Aug;187:193–202. doi: 10.1016/j.socscimed.2017.06.018 (PMC5529289; doi:10.1016/j.socscimed.2017.06.018)
Supplement: Table S2 [file mmc2.docx]

**Supplementary Table 2: Descriptive statistics within each cohort**

|  | **NCDS58** |  | **BCS70** |  | **T07** |  |
| --- | --- | --- | --- | --- | --- | --- |
|  | N | % | N | % | N | % |
|  | | | | | | |
| ***Transition Timing*** | | | | | | |
|  | | | | | | |
| *Leaving Full-time Education* | | | | | | |
| **16 or earlier** | 7,053 | 62.8 | 4,628 | 49.4 | 726 | 55.4 |
| **17-18** | 2,562 | 22.8 | 2,474 | 26.5 | 331 | 25.3 |
| **19-21** | 1,019 | 9.1 | 1,105 | 11.8 | 189 | 14.4 |
| **Not by 22** | 596 | 5.3 | 1,129 | 12.1 | 64 | 4.9 |
| *Entering Employment* |  |  |  |  |  |  |
| **16 or earlier** | 6,651 | 60.2 | 3,478 | 37.6 | 323 | 26.7 |
| **17-18** | 2,478 | 22.4 | 2,989 | 32.3 | 553 | 45.8 |
| **19-21** | 1,090 | 9.9 | 1,280 | 13.8 | 212 | 17.5 |
| **Not by 22** | 838 | 7.6 | 1,505 | 16.3 | 120 | 9.9 |
| *Entering Cohabitation* |  |  |  |  |  |  |
| **18 or earlier** | 1,272 | 11.4 | 80 | 9.1 | 87 | 9.5 |
| **19-21** | 3,469 | 31.0 | 2,088 | 23.6 | 199 | 21.7 |
| **Not by 22** | 6,454 | 57.7 | 5,967 | 67.3 | 631 | 68.8 |
| *First Child* |  |  |  |  |  |  |
| **21 or earlier** | 1,904 | 17.0 | 1,030 | 12.0 | 143 | 12.6 |
| **Not by 22** | 9,325 | 83.0 | 7,559 | 88.0 | 994 | 87.4 |
| *Leaving Parental Home* |  |  |  |  |  |  |
| **16 or earlier** | 723 | 6.4 | 226 | 2.8 | 31 | 2.5 |
| **17-18** | 2,860 | 25.5 | 1,655 | 20.3 | 96 | 7.6 |
| **19-21** | 3,644 | 32.4 | 2,401 | 29.5 | 351 | 27.8 |
| **Not by 22** | 3,862 | 34.4 | 3,870 | 47.5 | 783 | 62.1 |
|  |  |  |  |  |  |  |
| ***Early Adult Outcomes*** |  |  |  |  |  |  |
|  |  |  |  |  |  |  |
| *Daily Smoking* |  |  |  |  |  |  |
| **No** | 6,809 | 60.8 | 4,172 | 66.5 | 739 | 63.7 |
| **Yes** | 4,382 | 39.2 | 2,100 | 33.5 | 422 | 36.3 |
| *Heavy Drinking* |  |  |  |  |  |  |
| **No** | 8,366 | 74.5 | 5,046 | 78.5 | 712 | 61.6 |
| **Yes** | 2,856 | 25.5 | 1,384 | 21.5 | 444 | 38.4 |
|  |  |  |  |  |  |  |
| ***Background Confounders*** |  |  |  |  |  |  |
|  |  |  |  |  |  |  |
| *Gender* |  |  |  |  |  |  |
| **Male** | 5,620 | 50.0 | 4,485 | 47.9 | 687 | 48.1 |
| **Female** | 5,610 | 50.0 | 4,879 | 52.1 | 742 | 51.9 |
| *Parental Occupational class* |  |  |  |  |  |  |
| **Non-Manual** | 4,595 | 50.8 | 3,907 | 66.0 | 853 | 60.5 |
| **Manual** | 4,448 | 49.2 | 2,014 | 34.0 | 556 | 39.5 |
| *Parental Education* |  |  |  |  |  |  |
| **Post-16 education** | 1,534 | 16.5 | 1,900 | 31.1 | 495 | 35.2 |
| **Left at 16 or before** | 7,756 | 83.5 | 4,205 | 68.9 | 912 | 64.8 |
| *Household Income* |  |  |  |  |  |  |
| **Top and middle tertile** | 5,110 | 68.4 | 3,683 | 69.9 | 907 | 67.5 |
| **Bottom tertile** | 2,356 | 31.6 | 1,585 | 30.1 | 437 | 32.5 |
| *Family Structure* |  |  |  |  |  |  |
| **Single Parent** | 750 | 8.0 | 464 | 9.8 | 188 | 13.5 |
| **Couple Parents** | 8,658 | 92.0 | 4,251 | 90.2 | 1,207 | 86.5 |
| *Parental Smoking* |  |  |  |  |  |  |
| **Non-smokers** | 2,633 | 28.1 | 3,537 | 42.5 | 381 | 28.8 |
| **Smoking parent(s)** | 6,723 | 71.9 | 4,790 | 57.5 | 941 | 71.2 |
| *Parental Drinking* |  |  |  |  |  |  |
| **None to moderate** | 9,412 | 99.0 | 5,539 | 68.2 | 1,090 | 83.0 |
| **Heavy** | 95 | 1.0 | 2,588 | 31.8 | 223 | 17.0 |
| *Adolescent Smoking* |  |  |  |  |  |  |
| **Less than daily** | 7,028 | 73.9 | 4,583 | 81.5 | 1,208 | 84.9 |
| **Daily** | 2,480 | 26.1 | 1,040 | 18.5 | 215 | 15.1 |
| *Adolescent Drinking* |  |  |  |  |  |  |
| **Less than weekly** | 5,130 | 53.8 | 2,630 | 47.3 | 1,346 | 94.4 |
| **Regular (weekly)** | 4,403 | 46.2 | 2,933 | 52.7 | 80 | 5.6 |
| *Adolescent Distress* |  |  |  |  |  |  |
| **No or few symptoms** | 8,048 | 83.3 | 3,070 | 71.7 | 1,146 | 85.2 |
| **Symptomatic** | 1,612 | 16.7 | 1,214 | 28.3 | 199 | 14.8 |
| *School Performance* |  |  |  |  |  |  |
| **Taking less than 7 exams** | 4,642 | 48.3 | 1,007 | 32.7 | 783 | 55.2 |
| **Taking 7 or more exams** | 4,978 | 51.7 | 2,074 | 67.3 | 636 | 44.8 |
|  |  |  |  |  |  |  |
