# Supplementary material for: Causal effects of transitions to adult roles on early adult smoking and drinking: Evidence from three cohorts
Source: Soc Sci Med. 2017 Aug;187:193–202. doi: 10.1016/j.socscimed.2017.06.018 (PMC5529289; doi:10.1016/j.socscimed.2017.06.018)
Supplement: Table S3 [file mmc3.docx]

##### Supplementary Table 3: Model fit statistics for models with different numbers of classes

| **Number of Classes** | **Log-likelihood** | **AIC** | **BIC** | **Entropy** |
| --- | --- | --- | --- | --- |
| *NCDS58* |  |  |  |  |
| 2 | -50583.78 | 101217.57 | 101403.48 | 1.000 |
| 3 | -46954.15 | 93984.30 | 94266.88 | 0.975 |
| 4 | -44318.67 | 88739.35 | 89118.61 | 0.979 |
| 5 | -42809.06 | 85746.09 | 86222.02 | 0.966 |
| 6 | -41874.85 | 83903.70 | 84476.30 | 0.970 |
| 7 | -41238.94 | 82657.89 | 83327.17 | 0.968 |
| 8 | -41056.91 | 82319.82 | 83085.77 | 0.970 |
| *BCS70* |  |  |  |  |
| 2 | -50530.30 | 101110.60 | 101295.94 | 0.996 |
| 3 | -47146.24 | 94368.47 | 94650.19 | 0.940 |
| 4 | -45544.76 | 91191.52 | 91569.61 | 0.933 |
| 5 | -44084.31 | 88296.63 | 88771.10 | 0.941 |
| 6 | -43550.52 | 87255.03 | 87581.18 | 0.919 |
| 7 | -43136.47 | 86452.94 | 87120.16 | 0.891 |
| 8 | -43006.81 | 86219.61 | 86983.21 | 0.897 |
| *T07* |  |  |  |  |
| 2 | -4832.32 | 9714.65 | 9846.27 | 0.911 |
| 3 | -4627.95 | 9331.90 | 9531.96 | 0.778 |
| 4 | -4519.51 | 9141.01 | 9409.51 | 0.818 |
| 5 | -4420.68 | 8969.37 | 9306.31 | 0.837 |
| 6 | -4377.68 | 8909.36 | 9314.74 | 0.756 |
| 7 | -4356.28 | 8892.55 | 9366.38 | 0.765 |
| 8 | -4347.56 | 8901.12 | 9443.38 | 0.755 |
| *All Cohorts Combined* | | | | |
| 2 | -107842.67 | 215735.33 | 215939.69 | 0.994 |
| 3 | -101519.01 | 203114.01 | 203424.63 | 0.953 |
| 4 | -96296.32 | 192694.64 | 193111.53 | 0.943 |
| 5 | -93759.20 | 187646.41 | 188169.56 | 0.949 |
| 6 | -91762.83 | 183679.66 | 184309.08 | 0.935 |
| 7 | -90721.68 | 181623.36 | 182359.05 | 0.930 |
| 8 | No models converged within 1,000 iterations | | | |
|  |  | | | |
