# Supplementary material for: Causal effects of transitions to adult roles on early adult smoking and drinking: Evidence from three cohorts
Source: Soc Sci Med. 2017 Aug;187:193–202. doi: 10.1016/j.socscimed.2017.06.018 (PMC5529289; doi:10.1016/j.socscimed.2017.06.018)
Supplement: File S4 [file mmc4.docx]

**Supplementary File 4: Analyses with full un-truncated weights**

This supplementary file contains results using the full un-truncated weights. Figure 4-1 shows covariate balance post-weighting (pre-weighting balance is displayed in Figure 3 in the main article). In contrast to the truncated weights used in the main article, NCDS58 and BCS70 were balanced on all covariates for all early transitions groups. Where this balance was not replicated with the truncated weights early transition group members may be so different in terms of their background characteristics from those in *Tertiary Education* that this balance was only achieved by relying heavily on a small number of cases (i.e. having very high weight values for the top 5%). For T07 the full weights seem to have actually introduced some bias, with the *Early Adult* group now less likely than those in *Tertiary Education* to have single parents or to smoke in adolescence. This group also retained some imbalance on parental heavy drinking in this cohort. For these reasons we preferred to present results with truncated weights, even if some imbalance on covariates was retained.

Tables 4-1 and 4-2 show ORs and 95% confidence intervals for early adult smoking and heavy drinking respectively. In comparison to the main results, the weighted associations were generally more attenuated and had wider confidence intervals. Specifically, significant associations between early transition group membership and early adult smoking in BCS70 were not replicated with the full weights, nor were associations between smoking and membership of the *Early Adult* group replicated for any cohort with the full weights. For drinking, associations with less heavy drinking among the *Early Adult, Late Adolescent Work* and *Early Work then Family* groups were not replicated with the full weights for NCDS58 (but were in BCS70).


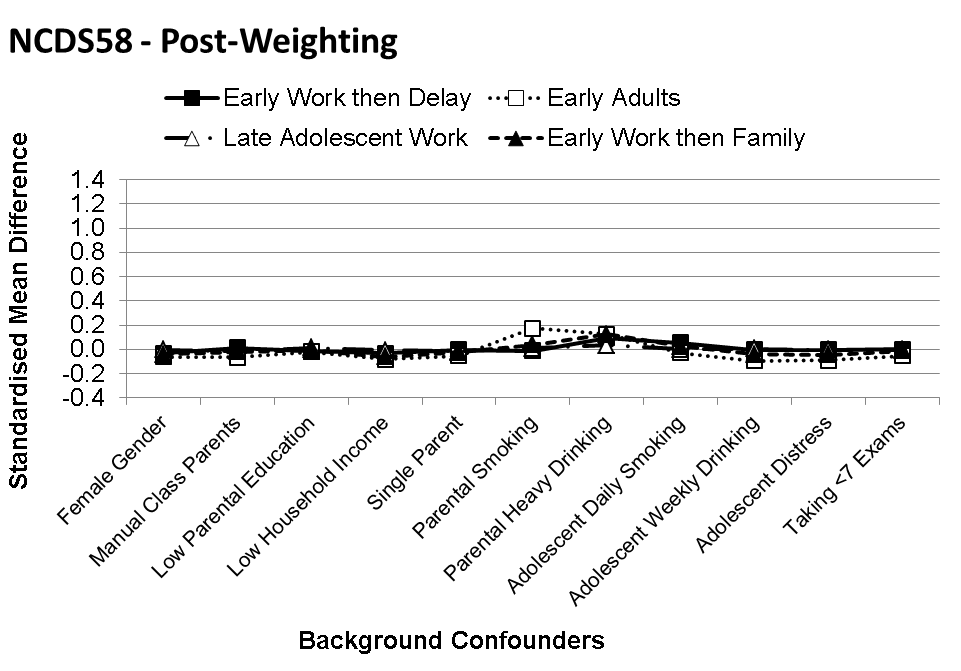


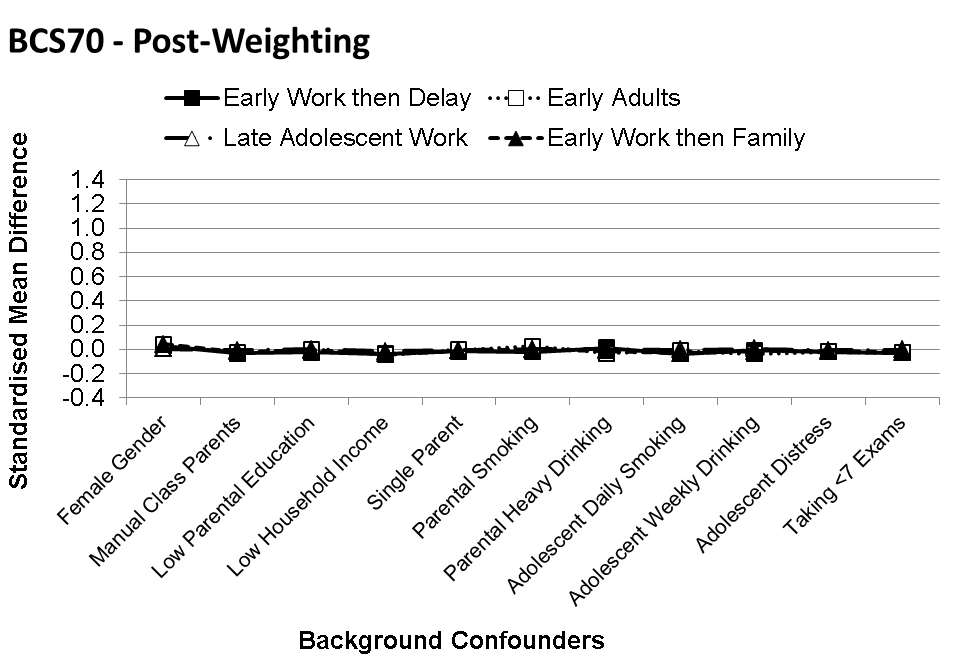

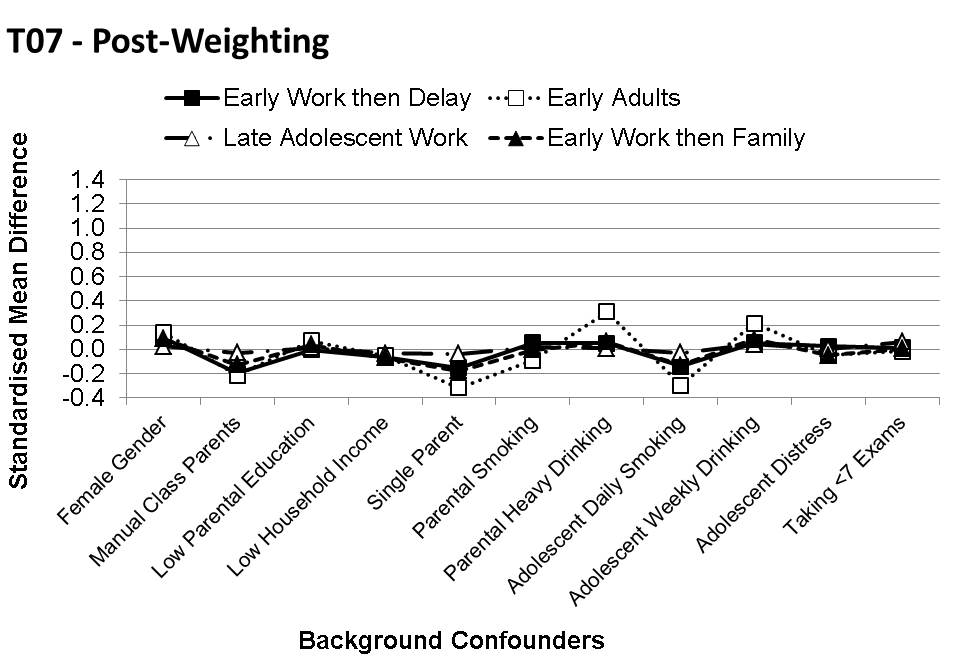


**Figure 4-1: Standardised Mean Differences on Background Confounders after Weighting (comparing to the *Tertiary Education* group)**

**Table S4-1: ORs for early adult smoking**

|  | **Pre-Weighting** | | | **Post-Weighting** | | |  |
| --- | --- | --- | --- | --- | --- | --- | --- |
|  | OR | 95% CI | P-value | OR | 95% CI | P-value | % difference |
|  | | | | | | | |
| *Early Work then Delay (ref: Tertiary education)* | | | | | | | |
| **NCDS58** | 2.78 | 2.43-3.18 | <0.001 | 1.85 | 1.24-2.78 | <0.001 | -52 |
| **BCS70** | 1.94 | 1.68-2.25 | <0.001 | 1.15 | 0.92-1.44 | 0.230 | -84 |
| **T07** | 1.76 | 1.26-2.46 | 0.001 | 0.83 | 0.43-1.62 | 0.592 | -122 |
| *Early Adult (ref: Tertiary education)* | | | | | | | |
| **NCDS58** | 4.91 | 4.16-5.79 | <0.001 | 1.98 | 0.72-5.48 | 0.186 | -75 |
| **BCS70** | 3.31 | 2.67-4.11 | <0.001 | 1.28 | 0.87-1.89 | 0.216 | -88 |
| **T07** | 4.72 | 2.96-7.53 | <0.001 | 1.53 | 0.53-4..47 | 0.435 | -86 |
| *Late Adolescent Work (ref: Tertiary education)* | | | | | | | |
| **NCDS58** | 1.66 | 1.45-1.90 | <0.001 | 1.41 | 1.17-1.70 | <0.001 | -38 |
| **BCS70** | 1.31 | 1.13-1.52 | <0.001 | 1.08 | 0.92-1.28 | 0.328 | -73 |
| **T07** | 0.90 | 0.60-1.34 | 0.599 | 0.75 | 0.46-1.21 | 0.232 | +149 |
| *Early Work then Family (ref: Tertiary education)* | | | | | | | |
| **NCDS58** | 3.25 | 2.81-3.75 | <0.001 | 1.89 | 1.09-3.31 | 0.024 | -60 |
| **BCS70** | 2.33 | 1.93-2.82 | <0.001 | 1.15 | 0.89-1.53 | 0.350 | -89 |
| **T07** | 1.84 | 1.18-2.88 | 0.007 | 1.18 | 0.58-2.38 | 0.645 | -79 |
|  |  |  |  |  |  |  |  |

**Table S4-2: ORs for early adult heavy drinking**

|  | **Pre-Weighting** | | | **Post-Weighting** | | |  |
| --- | --- | --- | --- | --- | --- | --- | --- |
|  | OR | 95% CI | P-value | OR | 95% CI | P-value | % difference |
|  | | | | | | | |
| *Early Work then Delay (ref: Tertiary education)* | | | | | | | |
| **NCDS58** | 1.47 | 1.29-1.67 | <0.001 | 1.60 | 1.16-2.20 | 0.004 | +27 |
| **BCS70** | 0.93 | 0.81-1.07 | 0.330 | 0.86 | 0.68-1.07 | 0.177 | +112 |
| **T07** | 1.35 | 0.99-1.84 | 0.061 | 1.80 | 0.92-3.54 | 0.088 | +130 |
| *Early Adult (ref: Tertiary education)* | | | | | | | |
| **NCDS58** | 0.46 | 0.38-0.56 | <0.001 | 1.25 | 0.59-2.66 | 0.557 | -147 |
| **BCS70** | 0.41 | 0.30-0.55 | <0.001 | 0.51 | 0.32-0.81 | 0.005 | -18 |
| **T07** | 0.67 | 0.41-1.10 | 0.112 | 2.12 | 0.58-7.83 | 0.258 | -442 |
| *Late Adolescent Work (ref: Tertiary education)* | | | | | | | |
| **NCDS58** | 0.85 | 0.73-0.98 | 0.022 | 0.94 | 0.79-1.11 | 0.437 | -58 |
| **BCS70** | 0.70 | 0.60-0.82 | <0.001 | 0.79 | 0.66-0.95 | 0.012 | -31 |
| **T07** | 1.13 | 0.78-1.64 | 0.502 | 1.21 | 0.78-1.86 | 0.393 | +54 |
| *Early Work then Family (ref: Tertiary education)* | | | | | | | |
| **NCDS58** | 0.65 | 0.56-0.75 | <0.001 | 0.96 | 0.64-1.44 | 0.843 | -15 |
| **BCS70** | 0.53 | 0.43-0.65 | <0.001 | 0.56 | 0.41-0.78 | 0.001 | -7 |
| **T07** | 0.74 | 0.47-1.17 | 0.197 | 1.66 | 0.86-3.23 | 0.132 | -357 |
|  |  |  |  |  |  |  |  |
